# Relationships among Retinal Nonperfusion, Neovascularization, and Vascular Endothelial Growth Factor Levels in Quiescent Proliferative Diabetic Retinopathy

**DOI:** 10.3390/jcm9051462

**Published:** 2020-05-13

**Authors:** Ho Ra, Jae Hyun Park, Jin Uk Baek, Jiwon Baek

**Affiliations:** 1 Department of Ophthalmology, College of Medicine, The Catholic University of Korea, Seoul 06591, Korea; raho@catholic.ac.kr (H.R.); pjhhu@naver.com (J.H.P.); winwinbjw@naver.com (J.U.B.); 2 Department of Ophthalmology, Bucheon St. Mary’s Hospital, College of Medicine, The Catholic University of Korea. #327 Sosa-ro, Wonmi-gu, Bucheon, Gyeonggi-do 14647, Korea

**Keywords:** proliferative diabetic retinopathy (PDR), nonperfusion (nonperfusion; NP), neovascularization (NV), NVD, VEGF

## Abstract

Purpose: To investigate the relationships among the retinal nonperfusion (NP) area, neovascularization (NV) area, and aqueous humor vascular endothelial growth factor (VEGF) levels in quiescent proliferative diabetic retinopathy (PDR). Methods: Forty-seven eyes from 47 patients with treatment-naïve PDR that did not show macular edema or vitreous hemorrhage were enrolled. NP area, NV number, and NV area were quantitatively measured using ultra-widefield fluorescein angiography in an automated manner. Aqueous humor VEGF level was measured using a bead assay. Results: The NP areas of the total, posterior pole, peripheral retinae, and NV area positively correlated with each other (all *p* < 0.034). NV number correlated with total NP area, peripheral NP area, and NV area (all *p* ≤ 0.001). VEGF levels were significantly positively correlated with total, posterior polar, and peripheral NP areas and NV area (r = 0.575, 0.422, 0.558, and 0.362, respectively; all *p* ≤ 0.012). In eyes with NV in the disc area, the VEGF level was higher compare to eyes without NV in the disc area (208.89 ± 192.77 pg/mL vs. 103.34 ± 132.66, *p* = 0.010). A multiple linear regression model using NP area, NV area, and NVD demonstrated good prediction for VEGF level (R2 = 0.417, *p* < 0.001) and revealed a significant contribution of the peripheral NP area in predicting the VEGF level (β = 0.497, *p* = 0.002). Conclusions: Aqueous humor VEGF levels in quiescent PDR eyes were associated with NP and NV areas, which had positive correlations with each other. In addition, the NP area of the peripheral retina was the most important predictor of VEGF level.

## 1. Introduction

Diabetic retinopathy (DR) is one of the most important complications in diabetic patients. The pathophysiology of DR is a highly complex process involving hyperglycemia, leukostasis, ischemia, and inflammation [1]. Upregulation of angiogenic cytokines and inflammatory mediators by metabolic disturbances is considered a core mechanism causing a chain of pathological processes in DR [2]. Proliferative diabetic retinopathy (PDR), the late stage of DR, is a major cause of severe visual loss [3]. 

The overall hypoxic state of the retina in DR is driven by capillary occlusion and retinal nonperfusion (NP). Nonperfusion areas (NPAs) are predominantly found in the mid-peripheral retina and are associated with DR severity [4,5,6]. Pathognomonic findings of PDR include formation of retinal neovascularization (NV), which can result in vitreous hemorrhage or tractional retinal detachment [7,8]. 

There seems to be a positive correlation between NP area and NV. Using the conventional fluorescein angiography (FA) of the macular area, Merin et al. [9] found that the frequency of NV increased with an increase in the size of the NP area. More recently, the association between peripheral NP areas and NV was reported using ultra-widefield fluorescein angiography (UWF FA) [5,10]. UWF FA highlighted the importance of peripheral lesions in DR [11,12]. Quantitative analyses using UWF FA revealed that a larger NP area was associated with the presence of posterior NVs and NV at the disc area (NVD) [13,14,15].

Vascular endothelial growth factor (VEGF) is known to play an important role in the progression of NP and the development of NV in DR [16,17,18]. Increased hyperpermeability, endothelial cell proliferation, and cell migration in PDR are largely mediated by VEGF [19]. VEGF injection was also reported to be sufficient to cause capillary occlusion with ischemia, thereby creating NP areas [20]. Experimental studies also revealed that VEGF can initiate the process of NV formation, which involves other pathways such as inflammation, cellular immunity, and downregulation of angiogenic inhibitors [21,22,23,24].

Despite the reported association between NP vs. NV, NP vs. VEGF, or NV vs. VEGF, direct quantitative correlation analysis among these markers has not yet been done. Therefore, the purpose of this study was to quantitatively investigate head-to-head relationships among NP areas, NV area and number, and VEGF levels in PDR eyes. 

## 2. Methods

This cross-sectional case series study was carried out in the Department of Ophthalmology at Bucheon St. Mary’s Hospital, Catholic University of Korea (Gyeonggi-do, Korea). This study was approved by the hospital’s Institutional Review Board and conducted according to the Declaration of Helsinki. Informed consent was obtained after the purpose and potential risks of the procedure were explained to each participant.

### 2.1. Patients

The study group consisted of all consecutive type 2 diabetic mellitus (DM) patients who underwent intravitreal bevacizumab injection for treatment-naïve quiescent PDR between August 2016 and September 2018 in the Department of Ophthalmology of Bucheon St. Mary’s Hospital. PDR was defined by manifested posterior segment NV [25]. NV in the disc (NVD) was determined if any NV were present inside a 1-disc diameter from the optic disc margin. Presence of diabetic macular edema (DME) can complicate interpretation of the result regarding VEGF level, while presence of pre- or subretinal hemorrhage or vitreous hemorrhage can interfere with the measurement of NP areas. Therefore, only quiescent PDR eyes were included for the study to ensure better clarity in the interpretation of the results. Exclusion criteria comprised the following: diabetic macular edema (DME) (i.e., central macular thickness (CMT) of 350 mm or less with intra- or subretinal fluids); eyes with hemorrhage; low-quality images (e.g., cataract or other media opacity, poor cooperation); PDR with anterior segment NV (i.e., NV at the iris or angle); any prior treatment for DR, including anti-VEGF therapy, intraocular or periocular steroids, laser photocoagulation, and vitrectomy; and the presence of any significant retinal pathology other than PDR.

Each patient underwent a complete ophthalmologic examination, including measurement of best corrected visual acuity (BCVA), slit-lamp examination, and dilated fundus examination. High-definition optical coherence tomography (HD-OCT) of the 6 × 6 mm area macular cube with the 512 × 128 protocol of Cirrus high-definition optical coherence tomography (Cirrus-HD 4000, Carl Zeiss Meditec, Jena, Germany) was used to measure CMT, which was generated by an internal algorithm, and to confirm the presence of fluids. Mydriatic UWF FA (Optos California P200DTx icg; Optos, Dunfermline, UK) was performed using a standard image view of 200° in a single capture. Demographic information was obtained, including age, gender, coexisting hypertension, systolic and diastolic blood pressure, duration of diabetes, random glucose level, glycated hemoglobin (HbA1c) level within the last three months, and serum levels of blood urea nitrogen (BUN) and creatinine (Cr).

### 2.2. Image Analysis

**Figure 1:**
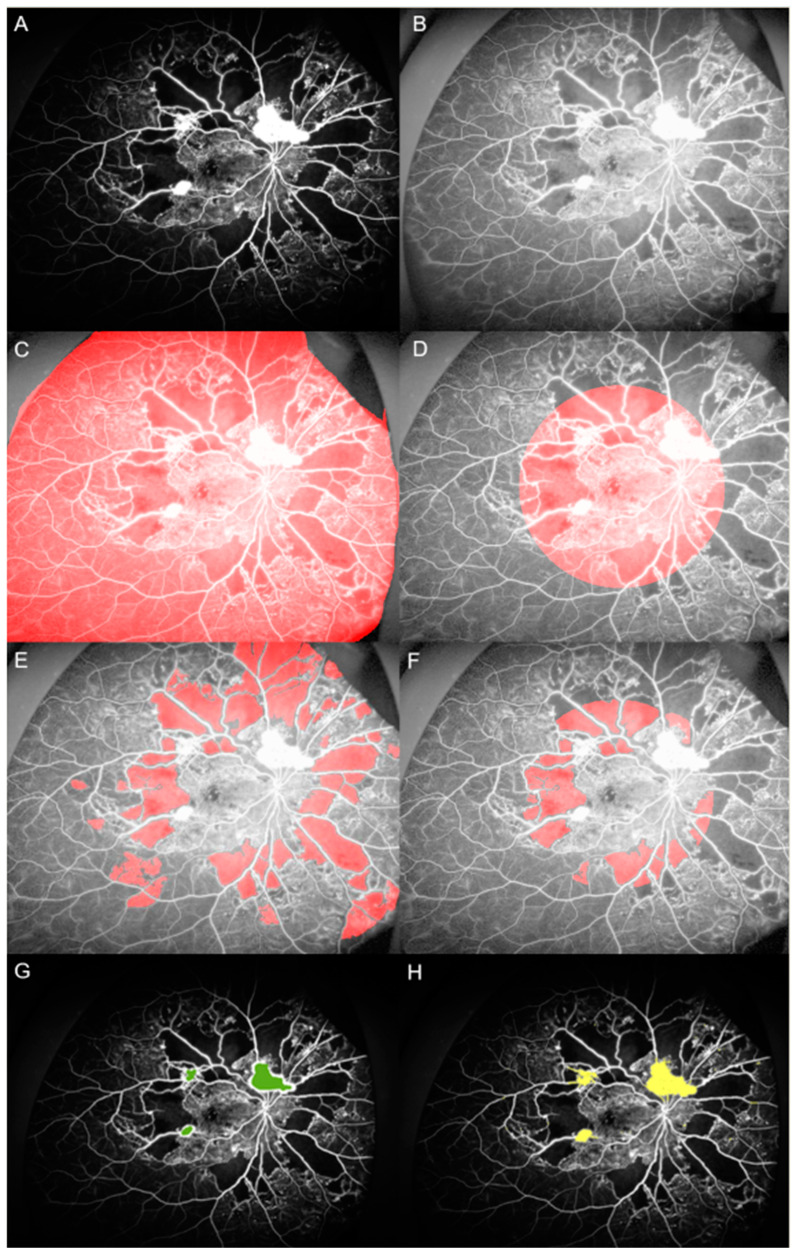
Assessment of nonperfusion area and neovascularization area with ultra-widefield fluorescein angiography. (**A**) Selected fluorescein angiography (FA) images were exported as an uncompressed Bitmap file. (**B**) The saved images were opened in MATLAB and preprocessed using homomorphic filtering for illumination correction. (**C**,**D**) Two regions of interest were manually selected: total gradable retina and the posterior pole. (**E**,**F**) The image was then segmented automatically and the nonperfusion area (NPA) was selected using a threshold of area properties. The selected NP area underwent a final manual modification. (**G**,**H**) Measurement of the neovascularization area was performed in a semiautomated manner using the Graph Cut and Active Contour functions in the MATLAB Image Segmenter application.

The nonperfusion area was defined as any area(s) on the retinal image with a size greater than the physiologic intercapillary distance and lacking fluorescein-filled capillaries. All NP patterns were included and no maximum size was prespecified [26]. Early- to mid-phase angiograms (between 45 s and 2 min) with minimal eyelid or eyelash artifacts were chosen for analysis of NP area. Areas of NP and NV were segmented and measured in an automated manner utilizing MATLAB R2019a (The MathWorks, Inc., Natick, MA, USA) (Figure 1A–F). The NP detection algorithm was largely based on the one proposed by Rasta et al. [27]. Briefly, the selected image was exported as an uncompressed Bitmap file. The saved images were opened in MATLAB and preprocessed using homomorphic filtering for illumination correction [28]. The preprocessed images were segmented automatically and NP areas were selected using a threshold of area properties (i.e., mean image intensity, uniformity, and entropy). The selected NP area underwent a final manual modification. NP areas were measured as both the total gradable retina and at the posterior pole. The posterior polar area was defined as a circular area centered on the fovea, with the radius determined as the distance from the fovea to the center of the optic disc. The peripheral NP area was defined as the difference of the total NP area minus the posterior pole NP area. 

NV was defined as a focal area of leakage on the angiogram, with a characteristic appearance of fine loops or a network of vessels [13]. UWF FA images taken prior to the appearance of leakage or showing minimal leakage (between 20–45 s) were chosen for analysis of numbers and areas of NVs to avoid erroneous enlargement of the measured area. This was also performed in an automated manner using the Graph Cut and Active Contour functions in the MATLAB Image Segmenter application (Figure 1G,H). The area of the optic disc was measured for each image. Measured NP and NV areas were divided into the disc area (DA). The presence of NVD was recorded. The measurement of NP areas, NV areas and number, and NVD was performed individually by two trained ophthalmologists (J.B. and J.W.B). If there was any disagreement in image tracings between the two graders, the senior retina specialist (J.B.) made the final decision.

### 2.3. Cytokine Analysis

Before the intravitreal bevacizumab injection, undiluted aqueous humor samples (0.1–0.2 mL) were aspirated by limbal paracentesis using a 30-gauge needle attached to a 1 cc syringe. The samples were placed immediately into sterile tubes and stored at −80 °C in a deep freezer until analysis. Concentrations of the VEGF cytokines in aqueous humor were determined using the MILLIPLEX^®^ MAP Human Cytokine/Chemokine Magnetic Bead Panel-Immunology Multiplex Assay (Millipore SAS, Molsheim, France).

### 2.4. Statistical Analysis

Statistical analyses were performed with SPSS for Windows (version 23.0.1; SPSS Inc., Chicago, IL, USA). Intraclass correlation coefficients (ICCs) were calculated for measurements of NP areas, NV area, and NV number. Pearson’s correlation analysis was used to determine the coefficients of correlation between clinical parameters, NP area, NV area and number, and cytokine concentrations after confirmation of a normal distribution. Linear regression analysis was performed using factors of statistical relevance. Automatic linear modelling was performed at the confidence level of 95%. A *p*-value < 0.05 was considered statistically significant.

## 3. Results

### 3.1. Baseline Demographic and Clinical Features

In total, 47 eyes from 47 type 2 DM patients with treatment-naïve quiescent PDR were enrolled. All patients were Korean and the mean age was 54.04 ± 9.33 years. In total, 49%were males and 79% had comorbid hypertension. The mean duration of DM was 14.98 ± 8.83 years. The mean corrected logMAR BCVA and CMT were 0.31 ± 0.37 and 279.96 ± 51.49 um, respectively. Baseline demographic and clinical features of study eyes are summarized in Table 1.

### 3.2. Nonperfusion Areas, Neovascularization Areas, and Numbers 

The mean NP areas of the total, posterior polar, and peripheral retinae were 73.49 ± 61.63 DA, 4.69 ± 5.42 DA, and 68.80 ± 59.35 DA, respectively. Of the total NP areas, 93.62% were located in the peripheral retina. The mean NV area was 2.91 ± 4.06 DA. The mean number of NVs was 4.59 ± 4.3. NVD was observed in 49% of the eyes (Table 2). 

The NP areas of the total, posterior pole, peripheral retinae, and NV area positively correlated with each other (all *p* < 0.034). The NV number demonstrated positive correlations with the total NP area, peripheral NP area, and NV area (all *p* ≤ 0.001). The presence of NVD was associated with the NP area of the posterior pole and NV area (*p* = 0.030 and <0.001, respectively). The correlations between angiographic parameters are summarized in Table 3.

### 3.3. Relationships Among Nonperfusion Areas, Neovascularization Areas and Numbers, and VEGF

VEGF levels showed strong positive correlations with the total, posterior polar, peripheral NP areas, and NV area (r = 0.575, 0.422, 0.558, and 0.362, respectively; all *p* ≤ 0.012). In eyes with NV in the disc, the VEGF level was higher compare to eyes without NV in the disc (208.89 ± 192.77 pg/mL vs. 103.34 ± 132.66, *p* = 0.010, Table 4). No significant correlation was found between VEGF level and other clinical parameters, including age, sex, blood pressure, DM duration, BCVA, CMT, glucose level, BUN, Cr, and HbA1c (*p* = 0.361, 0.146, 0.714, 0.160, 0.296, 0.441, 0.739, 0.764, 0.247, and 0.769, respectively; Table 5). 

**Figure 2:**
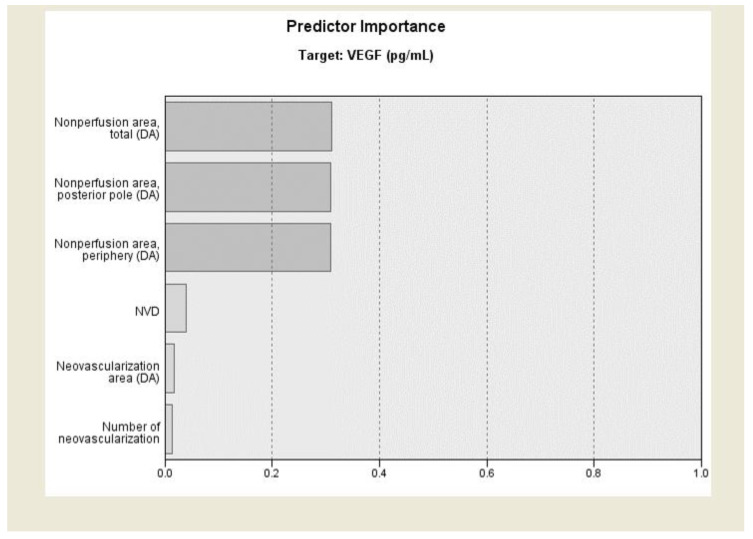
Predictive importance of each angiographic parameter to vascular endothelial growth factor. Automatic linear modelling demonstrated that the total, posterior polar, and peripheral retina NP areas were the most important predictors of aqueous VEGF levels.

Linear regression analysis, including NP areas, NV area, NV number, and NVD, revealed a good prediction of the VEGF level (R^2^ = 0.417, *p* < 0.001). The coefficients were significant for peripheral NP area (β = 0.497, *p* = 0.002). The result of automatic linear modelling revealed accuracy of 43.3% and demonstrated that the NP areas of total, posterior polar, and peripheral retina were the most important predictors of aqueous VEGF levels in a standard model. including all NP areas, NV area, and NV number (all *p* = 0.003, Figure 2).

## 4. Discussion 

Nonperfusion and NV are important targets in the treatment of PDR. VEGF is known to be involved in the development and progression of both NP and NV. In the current study, we analyzed quantitative correlations among NP areas, NV area and number, and VEGF levels in PDR eyes. The results showed positive correlations between NP areas, NVs, and aqueous humor VEGF levels. Furthermore, the importance of peripheral NV areas in the prediction of VEGF was emphasized by linear regression analysis.

Nonperfusion areas are not evenly distributed throughout the whole retina. Previous studies using conventional FA reported that the extent of the NP area is greatest in the midperipheral retina [26,29]. We utilized UWF FA to measure NP areas. Wessel et al. [12] reported that UWF images showed 3.9 times greater NP area and 1.9 times more NVs than seven standard field images of the early treatment diabetic retinopathy study. In this study, 93.62% of NP areas were located in the peripheral retina. This number is similar to that from other studies using UWF FA, showing that 86%–93% of the NP area was located in the mid- to far-periphery [4,15,26]. Nonperfusion areas of the total, posterior polar, and peripheral retinae showed positive correlations with each other. The high correlation between the peripheral NP and total NP can also be explained by the measurement method used in this study (i.e., total NP area minus posterior pole NP area). Although the proportion of posterior polar NP area to the total NP area seems small, it also reflects the global ischemic status of the PDR eye.

The NP areas of the total, posterior polar, and peripheral retina were correlated with NV area. An association between NP areas and risk for the development of PDR has been reported in recent studies using UWF FA. Baxter et al. [13] demonstrated that a NP threshold size greater than 23% of the retinal image is associated with posterior segment NV. Nicholson et al. [14] also suggested that at least 107.3 DA of NP are at risk of proliferative disease. This study adds the finding that the size of the NP area is also associated with the size of the NV area in PDR eyes. In addition to NV area, NV number was associated with the NP areas of the total retina. This trend was stronger for the NP area of the peripheral retina than that of the posterior pole. This may be explained by the distribution and absolute amount of NP areas, most of which were located in the peripheral retina. In this regard, the correlation between total and peripheral NP area was stronger than that of the posterior pole (coefficients were 0.997 for peripheral and 0.457 for posterior pole). Additionally, the presence of NVD was increased with larger posterior polar NP, suggesting the role of NP at the posterior pole in the development of NVD.

The VEGF level in the aqueous humor is strongly correlated with NP areas, regardless of the NP location. The correlation between VEGF level and NP areas was previously confirmed in eyes with DME [30,31]. To the best of our knowledge, this is the first study to evaluate the correlation between level of VEGF and NP area in PDR eyes without DME. Also, this study is the first to report the correlation between VEGF and NPAs using UWF FA, which may more precisely quantify the NPA of the whole retina. Because retinal ischemia is a major cause of VEGF upregulation in DR, the aqueous humor VEGF level may also correlate with the NP area [32,33].

VEGF is the most potent proangiogenic growth factor and is required in the DR process. Tolentino et al. injected VEGF into the vitreous cavity of monkeys and demonstrated that elevated levels of VEGF can cause retinal vascular leakage, hemorrhage, and telangiectasia [20,23]. A few eyes produced minimal peripheral NV, suggesting that pathologically increased VEGF might be a sufficient condition for NV in some cases. Nonetheless, automatic linear modelling in the current study revealed that NP areas can be more significant predictors of VEGF than NV. The positive correlation between VEGF level and NV area in the current study was not significant after adjusting for NP areas. NP areas, especially peripherally located ones, seem to have a stronger association with VEGF level than NV area or number. 

Since this is a retrospective cross-sectional study, we cannot confirm any causal relationship in the correlation between NP area and VEGF level. Nonetheless, the results of our study can be interpreted with caution using information from previous studies. Considering the role of hypoxia or ischemia in the overexpression of VEGF through hypoxia-inducible factor-1 in a hyperglycemic state, it can be cautiously assumed that an ischemic state following the development of retinal capillary NP might have led to overproduction of VEGF, which will eventually lead to angiogenesis of NVs. In this cycle, reduction of the VEGF burden by anti-VEGF treatment may lead to regression of NVs and further progression of VEGF-related pathologic changes. This was evidenced in DR eyes treated with anti-VEGF for DME [34,35,36]. It has been reported that panretinal photocoagulation (PRP) reduces VEGF and leads to NV regression [37]. This might be the result of a decrease in the NP area following PRP. Since an anti-VEGF injection alone does not seem to reverse NP area [38], a combination of anti-VEGF to reduce VEGF and PRP to decrease NP area seems optimal when considering the relationship between NP, NV, and VEGF. This combination treatment showed an earlier, faster, and larger reduction in areas of active NV than PRP alone [39,40,41,42].

This study has several limitations. First, we collected aqueous humor samples but not vitreous samples in this study; however, evidence supports a significant correlation of cytokine levels between the vitreous and aqueous samples, suggesting that aqueous cytokines represent an intraocular inflammatory status [43]. Second, although study patients were followed-up in the endocrinology department of our hospital, details on medical treatments (such as treatment protocol, follow-up interval, and compliance) were not controlled. Therefore, associations with clinical parameters such as BUN, Cr, blood pressure, or HbA1c require further investigation. Third, only quiescent PDR eyes were included in the study, while the role of VEGF could be more important in active PDR. However, the result showed that VEGF level correlated with NP area even in quiescent PDR. Further studies to reveal the link between quiescent and active PDR are warranted. Furthermore, vascular changes at the posterior pole can be more precisely visualized using OCT angiography, and validation of our results regarding the posterior pole warrants further study using OCT angiography. Nonetheless, this study has its strength, in that it is the first study to complete a head-to-head analysis of the relationships between NP, NV, and VEGF levels using UWF imaging and automated methods for imaging analysis.

In conclusion, the NP areas of the total, posterior pole, peripheral retinae, and NV area were associated with each other in quiescent PDR eyes. Aqueous humor VEGF levels were associated with NP areas and NV area, and the NP area of the peripheral retina was the most potent predictor for VEGF level. The result of the study emphasizes the importance of NP areas in VEGF-related problems in PDR eyes. Further follow-up studies area warranted to provide more valuable information on NP area and anti-VEGF treatment.

## Figures and Tables

**Table 1:** Demographic and clinical features of enrolled subjects.

Demographic Features	Mean ± SD	Range
Age (years)	54.04 ± 9.33	36–76
Gender (male/female)	23/24 (49)	
Hypertension (n) (%)	37 (79)	
DM duration (year)	14.98 ± 8.83	1–50
BP systolic (mmHg)	141.43 ± 21.36	115–183
BP diastolic (mmHg)	78.57 ± 8.73	60–97
BCVA (LogMAR)	0.23 ± 0.34	0–1.70
Central macular thickness (um)	281.77 ± 41.22	203–367
Glucose (mg/dL)	226.88 ± 102.5	88–415
BUN (mg/dL)	15.36 ± 5.11	8.7–34.3
Cr (mg/dL)	0.95 ± 0.53	0.46–2.84
HbA1c (%)	8.6 ± 2.25	5.5–13.6

SD: standard deviation; DM: diabetes mellitus; BP: blood pressure, BCVA: best corrected visual acuity; LogMAR: logarithm of minimum angle of resolution; BUN: blood urea nitrogen; Cr: creatinine; HbA1c: glycated hemoglobin.

**Table 2:** Nonperfusion areas, neovascularization area, and number.

Angiographic Parameters	Mean ± SD	Range
Nonperfusion area, total (DA)	73.49 ± 61.63	0.18–225.96
Nonperfusion area, posterior pole (DA)	4.69 ± 5.42	0–25.19
Nonperfusion area, periphery (DA)	68.8 ± 59.35	0–218.41
Number of neovascularization	4.59 ± 4.3	1–18.5
Neovascularization area (DA)	2.91 ± 4.06	0.05–16.5
NVD (n) (%)	23 (49)	

DA: disc area; NVD: neovascularization in the disc.

**Table 3:** Correlation among nonperfusion areas, NV areas, and numbers.

		NP Area, Total (DA)	NP Area, Posterior Pole (DA)	NP Area, Periphery (DA)	NV Area (DA)	Number of NV	NVD
NP area, total (DA)	Pearson’s Correlation		0.457 **	0.997 **	0.436 **	0.476 **	0.243
	*p*-value		0.001	0.000	0.002	0.001	0.100
NP area, posterior pole (DA)	Pearson’s Correlation	0.457 **		0.383 **	0.310 *	0.043	0.317 *
	*p*-value	0.001		0.008	0.034	0.773	0.030
NP area, periphery (DA)	Pearson’s Correlation	0.997 **	0.383 **		0.425 **	0.490 **	0.223
	*p*-value	0.000	0.008		0.003	0.000	0.131
NV area (DA)	Pearson’s Correlation	0.436 **	0.310 *	0.425 **		0.615 **	0.629 **
	*p*-value	0.002	0.034	0.003		0.000	0.000
Number of NV	Pearson’s Correlation	0.476 **	0.043	0.490 **	0.615 **		0.295 *
	*p*-value	0.001	0.773	0.000	0.000		0.044
NVD	Chi-square	0.243	0.317 *	0.223	0.629 **	0.295 *	
	*p*-value	0.100	0.030	0.131	0.000	0.044	

NP: nonperfusion; DA: disc area; NV: neovascularization; NVD: neovascularization at disc *. Correlation significant at the 0.05 level (2-tailed). **. Correlation significant at the 0.01 level (2-tailed).

**Table 4:** Correlation between angiographic parameters and VEGF level.

Angiographic Parameters		NP Area, Total (DA)	NP Area, Posterior Pole (DA)	NP Area, Periphery (DA)	NV Area (DA)	Number of NV	NVD
VEGF (pg/mL)	Pearson’s Correlation	0.575 **	0.422 **	0.558 **	0.362 *	0.210	0.382 **
	Sig. (2-tailed)	0.000	0.003	0.000	0.012	0.156	0.008

NP: nonperfusion; DA: disc area; NV: neovascularization; NVD: neovascularization at disc; VEGF: vascular endothelial growth factor. *. Correlation significant at the 0.05 level (2-tailed). ** Correlation significant at the 0.01 level (2-tailed).

**Table 5:** Correlation between clinical factors and VEGF level.

Clinical Factors	VEGF (pg/mL)	
	Pearson’s Correlation	*p*-Value
Age	−0.136	0.361
Gender	−0.215	0.146
HBP	0.055	0.714
BP, systolic	0.095	0.550
BP, diastolic	0.256	0.102
DM duration	−0.221	0.160
BCVA	0.156	0.296
CMT	0.115	0.441
Glucose	−0.054	0.739
BUN	0.048	0.764
Cr	−0.187	0.247
HbA1c	0.048	0.769

HBP: hypertension; BP: blood pressure; DM: diabetes mellitus; BCVA: best corrected visual acuity; CMT: central macular thickness; BUN: blood urea nitrogen; Cr: creatinine; HbA1c: glycated hemoglobin.
